# Seroepidemiologic Study Designs for Determining SARS-COV-2 Transmission and Immunity

**DOI:** 10.3201/eid2609.201840

**Published:** 2020-09

**Authors:** Hannah Clapham, James Hay, Isobel Routledge, Saki Takahashi, Marc Choisy, Derek Cummings, Bryan Grenfell, C. Jessica E. Metcalf, Michael Mina, Isabel Rodriguez Barraquer, Henrik Salje, Clarence C. Tam

**Affiliations:** National University of Singapore Saw Swee Hock School of Public Health, Singapore (H. Clapham, C.C. Tam);; Harvard University T.H. Chan School of Public Health, Boston, Massachusetts, USA (J. Hay, M. Mina);; University of California San Francisco EPPIcenter Program, San Francisco, California, USA (I. Routledge, S. Takahashi, I. Rodriguez-Barraquer);; University of California San Francisco Department of Medicine, San Francisco (I. Routledge, S. Takahashi, I. Rodriguez-Barraquer);; Oxford University Clinical Research Unit–Vietnam, Ho Chi Minh City, Vietnam (M. Choisy);; University of Florida Department of Biology, Gainesville, Florida, USA (D. Cummings);; Princeton University, Princeton, New Jersey, USA (C.J.E. Metcalf, B. Grenfell);; University of Cambridge, Cambridge, UK (H. Salje);; London School of Hygiene and Tropical Medicine (C.C. Tam)

**Keywords:** COVID-19, coronavirus disease, SARS-CoV-2, severe acute respiratory syndrome coronavirus 2, viruses, respiratory infections, zoonoses, immunity, study design, seroepidemiologic

## Abstract

Serologic studies are crucial for clarifying dynamics of the coronavirus disease pandemic. Past work on serologic studies (e.g., during influenza pandemics) has made relevant contributions, but specific conditions of the current situation require adaptation. Although detection of antibodies to measure exposure, immunity, or both seems straightforward conceptually, numerous challenges exist in terms of sample collection, what the presence of antibodies actually means, and appropriate analysis and interpretation to account for test accuracy and sampling biases. Successful deployment of serologic studies depends on type and performance of serologic tests, population studied, use of adequate study designs, and appropriate analysis and interpretation of data. We highlight key questions that serologic studies can help answer at different times, review strengths and limitations of different assay types and study designs, and discuss methods for rapid sharing and analysis of serologic data to determine global transmission of severe acute respiratory syndrome coronavirus 2.

Serologic studies are crucial for understanding current and future dynamics of the coronavirus disease (COVID-19) pandemic. In the past few months, much discussion about serologic studies and key issues with their design and interpretation has occurred. In this article, we discuss the questions that could be answered with these studies at different points in the epidemic and summarize the features and issues regarding study design, implementation of studies during an ongoing epidemic, and interpretation of the results. Discussion on the use of severe acute respiratory syndrome coronavirus 2 (SARS-CoV-2) serologic studies has largely focused on 2 questions: first, what proportion of a population has been infected?; and second, what proportion of a population is immune to disease or infection?

First, for infections that elicit detectable antibody responses, serologic studies can detect past infection regardless of clinical symptoms. This capability is useful for understanding the extent of past transmission (Figure 1, panel A). By linking this information with data on symptomatic cases, severe disease, and death in the same population, these studies can provide information on asymptomatic proportion, and the ratio of infections to severe cases and deaths (i.e., infection fatality ratio). Such data are also useful for calibrating mathematical models.

**Figure 1:**
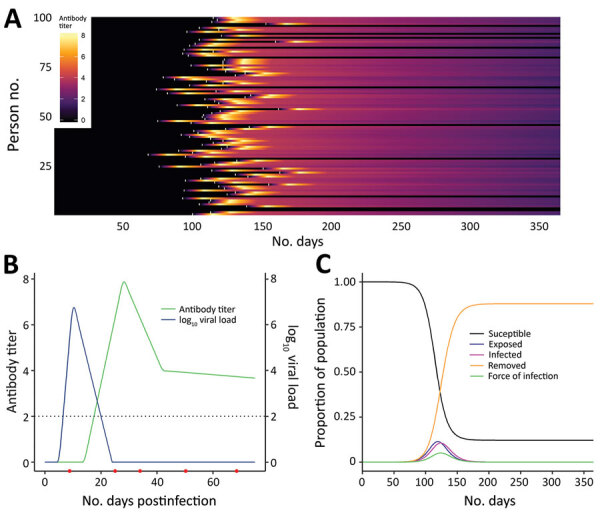
Link between severe acute respiratory syndrome coronavirus 2 infection dynamics and antibody levels in the population. A) Each line shows a person’s antibody titer. After infection, each person’s antibody levels undergo a dynamic process. A lag occurs from time of infection (white marks) to the generation of antibodies, which peaks several weeks postinfection and varies across persons depending on the time since infection and the parameters governing dynamics of the immune response. B) Antibody and virus dynamics in a person from time of infection. Frequent follow-up samples from the same person (indicated by red dots along the horizonal axis) would inform models of viral load and antibody kinetics. The dashed horizontal line represents the limit of detection of the assay. Early on, viral loads are more sensitive for diagnosing recent infection, whereas antibody titers become more sensitive once the humoral response is mounted and persons recover. C) Severe acute respiratory syndrome coronavirus 2 infections generated under an epidemic process (using a susceptible-exposed-infectious-removed model), modelling susceptible, exposed, infected, and recovered persons.

Second, if measured antibody responses correlate with protection, serologic studies can be used to measure the proportion of the population that is immune. This information can be used to guide control policies, help identify populations that are still susceptible to epidemics, target treatment or vaccination trials, and target vaccination when available. Although much discussion around use of serologic testing to inform persons of their serologic status has occurred, crucial distinctions exist between the use of serologic information to estimate population-level versus person-level immunity. Person-level immunity information is currently fraught with scientific, ethical, and legal uncertainties, which we do not address in this article.

## SARS-CoV-2 Antibody Response

Serologic studies will help answer these questions, but key unknowns persist regarding SARS-CoV-2 immunity and assay interpretation. Although estimating the proportion of the population that has been infected seems straightforward, careful consideration must be given to assay characteristics, the possibility for cross-reactivity with related coronaviruses, and the timing and magnitude of antibody responses. Timing and magnitude of antibody responses is particularly critical during a rapidly evolving epidemic, given the recency of infection for many persons.

Understanding population- or person-level protective immunity requires knowledge of how protective immunity is related to past SARS-CoV-2 infection, the extent to which antibody types or levels correlate with protection, and how long immunity lasts. Serologic assays detect presence of antibodies but generally do not establish whether those antibodies protect the person; neutralization assays are required to gain a deeper understanding of the functional role of antibodies in immune protection. Inferring protection from serologic tests is possible only after thresholds of protection have been established. Therefore, 3 additional intermediate questions need to be addressed to understand the benefits of conducting serologic studies and to interpret studies: 1) What is the antibody response observed after SARS-CoV-2 infections of different severity? 2) What is the extent of cross-reactivity in antibodies in different populations and in different assays? 3) Can we define a protective antibody response (a correlate of protection)?

A recent review summarized what is known regarding these questions for other coronaviruses (A.T. Huang et al., unpub. data, https://doi.org/10.1101/2020.04.14.20065771). For SARS-CoV-2, questions 2 and 3 can be answered with careful analysis of the types of seroepidemiologic studies proposed in this article, but question 1 requires a different type of study, one measuring antibody responses at multiple time points after acute infections of different severities (Figure 1, panel B). The first early studies on this subject suggest that some mild infections, or those in younger persons, might not lead to a measurable antibody response (F. Wu et al., unpub. data, https://doi.org/10.1101/2020.03.30.20047365), although more recent studies suggest that mild infections do lead to response (S. Fafi-Kremer et al., unpub. data, https://doi.org/10.1101/2020.05.19.20101832). This matter will be critical for inferring past infection from serologic tests and so requires continued study in different populations.

## Characteristics and Interpretation of Serologic Assays

New SARS-CoV-2 serologic assays emerge regularly (*1–3;* C. Sun et al., unpub data, https://doi.org/10.1101/2020.02.16.951723). These assays mainly fall into 3 categories: rapid tests; ELISA; and neutralization assays, such as plaque-reduction neutralization tests (PRNTs), microneutralization, or pseudovirus neutralization. Rapid, point-of-care tests generally use lateral flow immunochromatography and yield a qualitative (positive or negative) result. Despite their speed, ease of use, and amenability to mass screening, currently available rapid tests for SARS-CoV-2 have questionable accuracy. The World Health Organization (WHO) currently recommends that SARS-CoV-2 seroepidemiologic studies use IgG ELISA followed by confirmation of positive results with a PRNT (*4*). PRNT is recommended because it is more specific than other tests. Moreover, like other neutralization assays, PRNT provides quantitative information on antibody titers that inhibit viral infection, at least in vitro. However, PRNTs require dedicated laboratory training and facilities. They are more difficult to standardize and perform at large volume, and the tests must be performed in a Biosafety Level (BSL) 3 capacity laboratory, whereas ELISAs can be performed in BSL-2 laboratories (*5*). BSL-3 laboratories are not available everywhere. For global comparisons of serologic data based on different assays, understanding their comparability is key (*1*–*3*).

High sensitivity and specificity is desired for all assays but might be prioritized differently depending on the specific objective. For example, an assay that detects past infection with higher sensitivity (e.g., one that can detect antibodies at lower titers) might be insufficiently specific to determine who in the population is immune (e.g., if antibody titers are related to protective immunity). Specificity also might be a particular issue when infection prevalence is low (i.e., when the number of false-positive results could be substantial and even outnumber true-positive results).

Even at the person level, interpretation of serologic testing is time-dependent because detectable antibody responses might only appear ≈2–3 weeks after infection. In an ongoing epidemic, a large proportion of persons will be recently infected and therefore will be negative by serologic testing. Conversely, a substantial proportion of previously infected persons might have detectable virus for several weeks (Figure 1, panel C) (*6*). Tracking the proportion of the population infected over time might thus require use of PCR assays to detect recent infections, in addition to serologic assays, to minimize false-negative serologic test results obtained soon after infection. However, this approach would require the collection of additional respiratory or salivary samples, and a period in which infected persons are PCR-negative and have undetectable antibodies might occur. The additional use of different antibody subclasses (e.g., IgA and IgM) that might develop at different times during infection and can be measured in serum might help (A.T. Huang et al., unpub. data), although the timing of IgG and IgM might be similar (B. Berriman et al., unpub. data, https://doi.org/10.1101/2020.05.15.20103275). The extent to which serologic testing missing current or very recent infections affects results will depend on the prevalence in the population and growth rate. The extra effort to collect a swab specimen might be necessary at epidemic peak but less necessary at the tail end of an epidemic.

For different study types, the important assay characteristics are what sample is needed (e.g., serum, blood spot, nasopharyngeal swab, and nasal wash); where the assay can be performed (e.g., at home or in a laboratory); what resources, equipment, and reagents are needed; sample throughput and turnaround time; and cost. For public health, rapid and scalable approaches (e.g., point-of-care assays or laboratory testing of self-collected samples) are desirable. Such tests could also be useful for COVID-19 research in settings where restrictions on movement and social contact might limit the ability to collect samples. However, these methods need to be adequately validated before widespread use. The loss of information that comes with these tests might be offset by their ease of administration for some research questions but not for others.

## Seroepidemiologic Study Designs and Uses

We assessed 3 seroepidemiologic study designs: cross-sectional studies, cohort studies, and targeted population studies. We also address the question of at-home or on demand testing. We provide a description of each type, the questions they could help answer, and issues with representativeness and implementation during the pandemic (Table).

**Table:** Describing different study designs, questions they could answer, and issues with study design and execution during the coronavirus disease pandemic

Study type	Brief description	Questions study could answer	Issues with interpretation and representativeness	Issues with conducting during a pandemic
Cross-sectional	A sample of the population has serum samples collected at 1 time point	Background cross-reactivity (if started before pandemic); current proportion of population that have been infected; proportion of population that is immune (if a correlate of protection defined); infection fatality ratio (with information on cases or deaths in the same population)	For the different modes of collection (e.g., blood banks, residual sera, and volunteers), different issues can bias the sample included in the study that must be assessed	Blood banks might have fewer participants, residual sera studies in hospitals might have fewer samples or over representation of severe acute respiratory syndrome coronavirus 2 infections
Cohort	The same persons are followed up over time, with serum samples collected at regular intervals, and information on disease in intervening periods	Background cross-reactivity (if started before pandemic); ratio of asymptomatic to symptomatic infections; waning of antibody levels, correlates, and duration of protection; changes in infection dynamics over time	Attrition can make analysis and interpretation difficult, biases in which participants are retained across sampling rounds	Challenges in collecting and continuing cohort during outbreak; attrition
Targeted populations	Populations with particularly high exposures, such as those around index patients or healthcare workers, have serum samples taken either cross-sectionally or in a targeted cohort	Attack rates; ratio of asymptomatic to symptomatic infections; proportion of population infected, correlates, and duration of protection	Targeted populations because healthcare workers might have different infection exposure rates and intensity from the general population	Potentially logistically difficult to collect samples in household studies

### Cross-Sectional Studies

Cross-sectional studies measure prevalence of antibody responses in a sample of the population at a single time point. These studies might be repeated at multiple time points (a repeated cross-sectional design) but not necessarily from the same persons. What inferences can be made about the wider population depends largely on how representative the study sample is. Studies with representative simple- or cluster-based random sampling of the population are the gold standard but require extensive planning, resources, and community engagement. Many other potential sources of serum samples for cross-sectional serologic studies are available, including residual serum samples from patients undergoing medical investigations and blood donation banks. These studies can be conducted more rapidly on routinely collected samples and might have access to historically collected, preepidemic samples available for analysis. However, they require different considerations of representativeness; residual serum samples reflect persons who are generally more ill than the general population and might come with biases inherent in clinical testing criteria, whereas blood donors tend to be healthier and do not include children. Moreover, routinely available blood samples might lack information beyond basic demographics, such as geographic information, underlying conditions, or potential risk factors for infection that could affect the epidemiology and transmission of SARS-CoV-2.

Strict random sampling could also be relaxed by recruiting pragmatically through advertisements or from specific population groups but might be poorly representative and suffer from participation bias (e.g., if persons who think they have been previously exposed are more likely to participate). Invariably, a trade-off exists between ease of sampling and ease of interpretation, and studies employing more representative sampling strategies will yield more valuable information.

**Figure 2:**
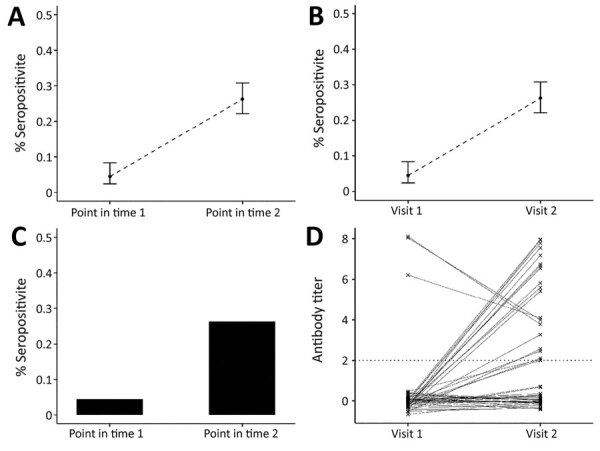
Link between severe acute respiratory syndrome coronavirus 2 infection dynamics and serologic analysis designs. A) Example of results from cross-sectional population study design, indicating percentage of study population who are seropositive at each sample time point. B) Example of results from a cohort study design: percentage of study population who are seropositive at each sample time point. The difference in the study designs is shown in panels C and D. C) In a cross-sectional design, we only know proportions in the population; however, panel D shows an example of each person’s antibody titers over time, illustrating that in a cohort study we can follow the dynamics of antibody response over time (e.g., the proportion who seroconvert and person-to-person variability).

Prepandemic samples can be used to determine background non–SARS-CoV-2 coronavirus serologic profiles in populations. Repeated cross-sectional studies during the pandemic can give information on the proportion of a population infected, immune, or both at different time points and potentially different age groups (Figure 2, panels A–C). If compared with surveillance data, results of such studies can be used to estimate a reliable denominator of number of infections in the population for calculating infection fatality ratio. Studies conducted in different locations, particularly ones using the same assay or, after standardization of results, using different assays, will enable assessment of spatial heterogeneity in transmission.

During the pandemic, social distancing measures might restrict the ability to collect serum samples. Studies of residual serum samples might also be affected by reductions in hospital visits by noncritical patients, which will skew samples collected toward those from patients with substantial disease or COVID-19 patients. Blood banks might also have fewer donors during this period.

### Cohort Studies

In cohort studies, the same persons are followed over time and samples collected periodically (Figure 2, panel D) (*7*,*8*). During the intervening period, or at the point of sample collection, information might be collected on symptoms, healthcare use, and potential risk factors for infection. Cohort studies provide rich information but are expensive and labor-intensive to conduct. These studies might be conducted in communities, among specific populations (e.g., healthcare workers or pregnant women), or for biobanking. Cohorts can suffer from attrition, which causes issues in analyzing and interpreting the results. Therefore, during the pandemic, assessing whether cohorts should be collecting samples from, or information on, cohort participants who have died might be warranted.

Long-standing cohort studies might have historic serum samples to determine preepidemic non–SARS-CoV-2 coronavirus serologic profiles. In the short term, cohort studies can determine the incidence of infection. Concurrent symptom surveillance and acute illness sampling within cohorts can add considerable information and help determine the ratio of asymptomatic to clinically apparent infections. However, because of limits in cohort size, the number of severe cases might be insufficient to precisely estimate ratios of infection to severe illness and death.

Cohort studies might be useful to determine correlates of protection and the duration and waning of immunity. Depending on the size, demographics, and geographic distribution of the cohort, these studies might provide information on the serologic profile in different population subgroups. However, in some cases, these cohorts might be geographically or demographically restricted, so extrapolation to the whole population might not be possible.

Using existing cohorts, after making adjustments to ensure the cohorts capture information relevant to COVID-19, obviates the need to set up new cohorts. However, ongoing cohort studies might face restrictions on data and sample collection during the outbreak, and innovation might be needed.

### Targeted Population Studies

Targeted population studies might be conducted by following populations with high infection risk or in whom infection has wider consequences, such as healthcare workers or households of case-patients. However, the exposure of healthcare workers might not be similar to that of the rest of the population. 

As well as being of use for understanding infection rates in this important population, samples from healthcare workers and household members could be used to determine correlates of protection by comparing antibody profiles and subsequent infections. Fewer constraints on such collections would exist during an outbreak because healthcare workers will be coming to healthcare settings, where this type of study could be conducted.

Early in the pandemic, household studies might be useful to understand the proportion of infections that are asymptomatic; they are also useful in understanding age-specific infection rates because household members will vary in age but presumably will have exposure to any infectious person in the household. An advantage of these studies is that household contacts of a case-patient have greater exposure to infection at that time and that person’s exposure within households would likely be fairly uniform. Similar to previous study designs, issues with collecting samples under movement restrictions might exist.

### Persons Getting Tested for Their Own Personal Knowledge

Although not an epidemiologic study design, allowing persons to undertake home serologic tests to determine if they have been infected has generated interest recently. If a positive test result correlates well with immunity, then such tests could help determine which persons are no longer susceptible to infection and can safely resume normal activities. These samples might not be representative because persons might be more likely to request testing if they think they have been infected or if a family member or contact has been infected, and certain demographics might be overrepresented or underrepresented. The addition of a research component to such assay deployments, where persons are recruited randomly, could help overcome the limitation of information derived from this means of collection and provide valuable data.

Current tests are not sufficiently accurate for this use, and questions remain about the relationship between seropositivity and magnitude and duration of protection. However, such information could still have public health value. With additional demographic data on persons being tested, this approach could give information about the proportion of the population that has been infected (with caveats regarding the representativeness of the tested population). This approach might be less limited by restrictions on movements because of COVID-19.

## Analytical Methods for Inferring Past Infection from Serologic Data

Recently, analytical methods for using serologic data to determine prevalence of past infection have progressed considerably, through an improved understanding of immunology and novel statistical methods. A substantial body of literature now exists on statistical and mathematical modeling methods for antibody data, alongside off-the-shelf software packages. Antibody levels vary over time for each infected person in a population experiencing a COVID-19 epidemic (Figure 1). Existing methods to analyze this process fall into 2 broad categories. In the first category are methods that reduce assay results into binary metrics of seropositivity (a single reading above a specified threshold) (Figure 2, panels A and C) or seroconversion (an increase between 2 time points for the same person above a threshold) (Figure 2, panels B and D). In the second category are methods that incorporate full information on the magnitude or time series of antibody assay results (Figure 1, panel C).

If SARS-CoV-2 infection is found to generate stable, consistent antibody dynamics after infection, then binary metrics are likely to give accurate estimates of seroprevalence, albeit with considerations of assay variability and the sensitivity of the chosen threshold (*9*,*10*). The attack rate and force of infection (Figure 1, panel B), measures of transmission intensity, can then be calculated with existing serocatalytic models and software packages (*11*,*12*), with statistical adjustments for the (known) sensitivity and specificity of the chosen assay and the time lag between infection and seropositivity (*11*–*15*).

However, if antibody kinetics after SARS-CoV-2 infection follow a more complicated trajectory, then models that capture additional immunologic mechanisms (e.g., timing of development, antibody waning, cross-reactivity with other pathogens, or variation in person-level responses) will be required (*16*,*17*). The importance of these variables will depend on SARS-CoV-2 immunology, properties of the chosen assay (*3*), and the antibody isotype (*1*,*18*) being measured. Combining results from different assays and isotypes might be a powerful approach.

Methods for accounting for bias in the way samples are collected, and therefore who is in the study, must also be used, bearing in mind that for some study designs, fully accounting for biases will not be possible. The sensitivity and specificity and the proportion of the population that is infected are important for interpreting results. Methods for estimating population infection prevalence from serologic data are being developed, and one such method is described by Larremore et al. (*19*)

## Analytical Methods to Infer Correlates of Protection

A recent review highlighted that, for the seasonal coronaviruses, only human challenge experiments have provided the level of data needed to identify a correlate of protection (A.T. Huang et al., unpub. data). Establishing a correlate of protection is difficult for any infectious disease, but challenges exist for novel pathogens, particularly during an outbreak with rapidly changing dynamics. When using the results from cohort studies as we have described, statistical analysis comparing preexposure immune responses in persons who subsequently have disease versus those that do not can provide information about whether an assay has characteristics that are useful for defining protective immunity or a correlate of protection. Similar work has been done for chikungunya and influenza viruses (*20*,*21*). However, potential confounders, such as differences in exposure risk over time, must be considered carefully in this type of analysis. Any measured level or correlate of protection will be specific to the assay used in the studies, as was the case for measles (*22*). Even for measles, where a correlate of protection has been used consistently, a recent review found little evidence to support the threshold used, suggesting this threshold needs refinement (*22*).

## Comparing and Extrapolating Serologic Data across Studies and Geographic Regions

Studies will be useful for understanding situations in a particular population, place, and time. An important consideration is whether results can give information about transmission or immunity in other groups. Age, underlying conditions, or a combination of both have been shown to affect infection outcome and therefore should be considered carefully when extrapolating from one population to another. For the asymptomatic proportion, differences in reporting and surveillance systems between places and over time mean extrapolation should be done with care.

The use of various assays in different places shows the need to determine whether building methods to compare results from different assays in different populations is possible. Quantifying intra- and inter-laboratory variability of the same assay and between-assay variability will therefore be crucial.

## Rapid Sharing and Comparative Analysis of Serologic Data

As proposed previously (*23*), rapid sharing and dissemination of serologic data are useful for clarifying infectious disease dynamics and have become even more vital given the urgency of questions in the current pandemic. Phylogenetic analysis has greatly benefited from development of the GISAID database (https://www.gisaid.org) to enable rapid sharing of genomic data and from platforms like Nextstrain (*24*) for rapid analysis. Rapid sharing would enable comparison of assays across populations (including comparing prepandemic samples for understanding cross-reactivity in different populations). Rapid sharing would also enable pooled analyses, comparison of parameters of transmission, and gauging of the effect of interventions across place and time. If a database were to be developed, a core set of data would need to be collected on each sample and on each study and assay. WHO has proposed that all such data be shared with WHO and has issued standardized protocols (*4*), but even more open sharing would also enable rapid analysis and decision making. The rapid sharing of tools specifically to analyze SARS-CoV-2 serologic studies will also be useful. Even if data sharing is not possible, as results of seroepidemiologic studies are released, they need to clearly show how subjects were recruited, what inclusion criteria and which assay were used, and how the analysis was conducted.

## Conclusions

Serologic studies at multiple stages of an epidemic could provide fundamental information for understanding the extent of past transmission, the current state of the epidemic, and future transmission. However, successful deployment of serologic testing will require optimization, validation, and proper interpretation of assays, which requires studies focused on these specific questions. Different types of epidemiologic study will be best and viable at different times during the outbreak and in different settings, and the biases of these study designs should be carefully taken into account in analysis and interpretation. Triangulation between multiple types of studies might also be of use. Current movement restrictions might constrain implementation of some study designs, so thought should be given to other study designs, although with consideration of their possible biases. The utility of serologic studies can be even greater if they are designed for optimal cross-location comparison. A platform to enable rapid data sharing, and therefore analyses across places and times, would also be very powerful.
